# Round the Hospitals

**Published:** 1920-03-06

**Authors:** 


### ROUND THE HOSPITALS.

Princess Mary attended a reception at the Mansion House on February 26 and received purses in aid of the funds of the London Fever Hospital. Mr. James C. Marshall presented a handsome sum collected from colleagues in the textile trades. Among the donors was the famous old English sheep-dog Rip, who was made much of by the Princess, though he modestly withdrew as soon as he had made his offering. The Lord Mayor presided and Lord Balfour of Burleigh, who has been

**ROUND THE HOSPITALS**—(*continued*).

president of the hospital for nearly twenty years, gave an interesting address explaining that this was the only voluntary hospital for infectious diseases in London. Lord Knutsford added a tribute to its unique services among those working in other hospitals. Nurses, he explained, were particularly liable to infection, and it was a matter of intense anxiety that the women who were risking their lives should have proper care when themselves in danger. Such cases are sent to the London Fever Hospital and are nursed back to health with every advantage skill and science can supply.

The King held an Investiture at Buckingham Palace on February 25, and personally conferred decorations on the following members of the nursing services:—Royal Red Cross, 1st Class: Miss Annie Gray, Q.A.I.M.N.S. (R.). Royal Red Cross, 2nd Class: Mrs. Mary Helm, Miss Edith Mason, Miss Florence Parsons, Miss Eliza Thomas, Miss Amy Waterman, and Miss Margaret Wolsey, Q.A.I.M.N.S. (R.); Miss Mary Bunting, Miss Annie Knox, Miss Ann Smith, and Miss Boone Turner, T.F.N.S.; Miss Beatrice Short, C.N.S.; Miss Bertha Collins, Mrs. Mary Noel-Jones, Miss Sarah Williamson, and Miss Margaret Woodward, Civil and War Hospitals; Miss Faith Allen, Miss Edith McCarthy, Mrs. Ethel Ramsbotham, and Mrs. Annie Thomas, V.A.D. All the above ladies, together with Miss A. B. Smith, R.R.C., were subsequently received at Marlborough House by Queen Alexandra.

At a second Investiture held last week, on February 27, the King personally conferred decorations as follows:—Bar to the Royal Red Cross: Miss Edith McCall-Anderson. Royal Red Cross, 1st Class: Miss Janey Gray and Miss Kate Worger, T.F.N.S. Royal Red Cross, 2nd Class: Miss Humphrey Jones, Q.A.I.M.N.S.; Misses Jessie Blyth, Jessie Cumming, Adelaide Eves, Jessie Hoskins, Anne Lee, Dorothy Taylor, Q.A.I.M.N.S. (R.); Mrs. Maude Gibson, Theresa Hayes, Marion Hearn, Maud Hall-Houghton, Rowener Lush, Agnes Watson, Sarah Youngman, T.F.N.S.; Mrs. Whyte, Mrs. Williamson, Miss Jessie Wilson, B.R.C.S.; Misses Sybil Grey and Kate Haywood, Civil and War Hospitals; Mrs. Warde-Aldane, Miss Winifred Clark, Viscountess Ednam, Misses Hilda Lyster, Dorothy Taylor, and Marjorie Williams. Queen Alexandra received all the above ladies, together with Dame Sydney Browne, R.R.C., after the Investiture at Marlborough House.

The formal opening of the Washington Ward at the Royal Free Hospital on February 24 by Princess Marie Louise, President of the Ladies' Association, was one of those occasions which illustrate the strong bond of sympathy which draws American and English women together in good works. This handsome maternity ward is the gift of the American Women's Club, and they have undertaken to maintain it, a magnificent free-will offering. The Princess's graceful thanks to the American ladies, through whose exertions the enterprise has been carried through, express in brief compass the sentiments of English women to their confrères in the United States. Should we not all echo her words, and convey to the American people that we welcome their co-operation with joy and friendship whenever and however they may come over to help us in our common tasks for the benefit of humanity?

We are requested to announce that the Nurses' Resettlement Department of the Ministry of Labour has removed from Curzon Street to 99 Queen's Gate, S.W.7. Though its labours have been considerably reduced they are as yet by no means at an end.

A useful handbook has been issued by the Ministry of Health giving a list of Maternity and Child-welfare Centres in England and Wales, with a complete index. The total number of centres amounts to 1,718, of which 351 are conducted by the county councils, 708 by local sanitary authorities, and 679 by voluntary societies. Large as the list may appear, there is room for a great expansion of this kind of effort. London, in particular, with but 203 centres must be considered very inadequately served considering that centres need to be brought within easy reach of the clientele if they are to prove really useful.

The news that Miss Cowlin, Organising Secretary of the College of Nursing, has been appointed Assistant-Director of the nursing division of the League of Red Cross Societies at Geneva, will be heard with very mixed feelings. On the one hand, her numerous friends will rejoice at her obtaining this influential post, so well suited to her talents and high aims. On the other hand, she will be a great loss to the College, where her work in organising the Local Centres testifies to her abilities. Miss Cowlin has had an interesting and varied career. She was trained at St. Bartholomew's Hospital, and had the advantage subsequently of taking a course in hospital economics at Teachers' College, Columbia University, New York. She served as a member of the T.F.N.S. for two and a-half years in the 1st London General Hospital, and since 1916 has been attached to the College of Nursing, first as assistant and latterly as Organising Secretary. She is a clear and persuasive speaker, instinctively trusted by those with whom she has to do.

New grants and allowances for war disabled nurses and their dependants have now come into force, and will bring great relief to many who are now exposed to harsh circumstances. The full text of the official announcement appears on p. 547. The new grants and allowances apply to members of all the regular military and naval nursing services or services admissible under the Royal Warrant. A supplementary or special allowance may be granted to a disabled nurse

**ROUND THE HOSPITALS—(*continued*).**

unable to maintain herself in her pre-war standard of comfort, provided that it does not bring the total pension up to more than £90 a year. Grants can be made to nurses to meet temporary distress or emergencies, and loans or recoverable advances may also be made in cases of necessity. A supplementary allowance may be obtained by the parent, brother or sister of a nurse who has lost her life owing to the war, in the event of such relative having been receiving regular support before or during the war from the nurse. When the nurse, by reason of disablement arising from service during the war, is unable adequately to maintain her children, or where a nurse dies in consequence of war service, allowances for maintenance and education, not exceeding those made under similar circumstances for the children of officers, may be granted, if the husband or widower of the nurse is dead, or unable to support the children. Applications for these grants or allowances should be headed S.G.O., and addressed to the Secretary, Special Grants Committee, Officers' Branch, Millbank House, Westminster.

---

The *Daily Telegraph* Fund for Nurses has now reached a total of 168,564 shillings. The contributions of the training-schools and of nurses themselves continue to witness to the desire of the nursing profession to be associated with Lord Burnham's generous efforts on their behalf. Among these we note a second instalment from the Nightingale School for Nurses; further subscriptions from London Centre of College of Nursing; Miss Lakeman and staff of Cane-hill Asylum; from matron and staff of Queen Mary's Hospital, Stratford; from staff of Military Hospital, Hounslow; from Bury Private Nursing Home, Lancs; from the Metropolitan Hospital; from the Infirmary, Plumstead; from Dundee Centre of College of Nursing; Burghmuir Hospital, Perth; Victoria Hospital, and Women's Hospital, Dundee; Infant Hospital, Broughty Ferry. The response from the officers' and sergeants' mess of various regiments continues to be very gratifying. Although the total is yet far from what the enthusiastic have in their minds, the cumulative effect of so many comparatively small contributions is very impressive. One can understand that some who have it in mind to crown the collection with the big sums for which the promotors appeal may be staying their hand for awhile to see what the comparatively poor can achieve as a token of gratitude to the nurses.

---

Miss Cummins, matron of the Liverpool Royal Infirmary is to be congratulated on the performance of an intensely disagreeable but most necessary public duty in prosecuting, with the chairman's approval, Miss Janet Foster, school nurse and health visitor at Rawtenstall for proffering a bogus eulogistic testimonial signed "E. Cummins," matron, Royal Infirmary, Liverpool, to the matron of the Lewisham Infirmary. Fortunately the matron at Lewisham wrote asking if Miss Cummins could recommend the applicant for the vacant post of sister. It then transpired Miss Foster was unknown at the Royal Infirmary, where she stated she had been trained and where she professed to have been sister, so recently as 1914, of *the* (*sic*) male surgical ward. The magistrate strongly upheld Miss Cummins' action, and fined Miss Foster £5, with the remark that she might consider herself very leniently dealt with. The incident shows, as Miss Cummins wisely points out, how essential it is that testimonials should be strictly verified and only written in exceptional cases.

---

A gratifying announcement was made at the annual meeting of the Central Council for District Nursing in London, held at the Offices of the Metropolitan Asylums Board with Sir William J. Collins in the Chair. No less a sum than £10,000 has been placed at the disposition of the Executive Committee by the British Red Cross Society. Its allocation has not yet been decided on, but as they were straitened for means during the past year it is a very welcome grant. The London Insurance Committee, with certain approved societies, has made a useful grant of £500 for nursing insured persons, and Mr. E. B. Turner stated that the Council hoped it was an earnest of a good deal more from the same direction, which would enable them to increase their nursing staff. There is no doubt that a wide extension of insurance nursing ought to result in securing very good posts for the nurses who undertake this class of work.

---

The influence which the Ministry of Health is already exerting on backward institutions is illustrated by a remark of the Chairman of the Braintree Board of Guardians. The infirmary is a small one, and the insufficient nursing staff has hitherto numbered only seven. The chairman informed the Board that the Ministry of Health had been pressing them very hard to increase the staff, and accordingly he proposed that a charge nurse be engaged at £45 to £50 a year with a junior assistant at £20 to £25. Admitting that nurses had been working too many hours he thought this was the minimum. Under the circumstances it does seem a pity that the Board should be content with the minimum. They have arrears to make up, and a third pair of new hands would ease the situation all round.

---

At a fire which broke out recently in the officers' mess of the British 82nd General Hospital at Constantinople, five patients were burned to death. It is reported that many more would have perished had it not been for the courage and resource of the English medical staff and nursing sisters, who devoted themselves to the task of clearing the wards with amazing speed and success. There were 600 military, naval, and Russian patients in the hospital, many of whom had to jump or be dropped into blankets from the windows. The hospital is in

**ROUND THE HOSPITALS—*(continued).***

the Turkish barracks near the German Embassy, and one wing has been entirely destroyed.

---

The word "Bedlam," one of the most common corruptions of Bethlehem, has haunted many a man and woman in the long past, from the institution's foundation in the thirteenth century, when six patients were removed by Royal command to its priory owing to the noise and racket they made. In his lecture before the Royal Society of Arts last week, the Rev. E. G. O'Donoghue, Chaplain to the Hospital, told the history of the institution and of its many dishonest and fraudulent stewards and medical superintendents in the Middle Ages. One Peter the Porter annexed many of the properties of the institution and of the patients, including chains of iron, bedclothing, manacles, and 10s. received from a woman patient for her half year's keep. His wife, Alice, kept a tavern at the hospital gates, where she sold beer at exorbitant prices to the patients, who could obtain it from her alone. Amongst the excellent slides shown by Mr. O'Donoghue was one representing the quarterly promenades when the public enjoyed teasing the unfortunate patients as much as a child enjoys the monkey-house to-day. They invariably brought with them liberal supplies of drink, with disastrous results. The "Bedlam" of to-day does much to illustrate the wonderful progress made during the last century in the treatment of the mentally sick, especially when it is remembered, as one of the audience pointed out, that less than one hundred years ago the northern coach stopped ten minutes at York for the passengers to make faces at the inmates of the asylum!

---

Steps ought certainly to be taken by the Central Midwives Board to procure the recognition of their certificate by the Cape Town registration authorities and safeguard their registered midwives from the unnecessary humiliation of being regarded in South Africa as outsiders. A case has recently come before the Liverpool Select Vestry of a lady trained at the Brownlow Hill Infirmary and fully certificated, who was refused registration as a trained nurse and midwife on going out to the Cape owing to objection being taken to her certificates. Some measure of reciprocity there must be in these matters, or even after the State Register has been established nurses will find themselves exposed to the annoyance of a demand for re-examination whenever they cross the boundaries of their own registration area. The Liverpool vestry are endeavouring to secure recognition of their certificates from the Colonial authorities, but that seems to us a very lame way of tackling the matter. It needs clearing up once for all by the C.M.B.

---

In recording staff resignations, the Willesden Urban District Council quotes the causes, which are interesting. Thus of nine probationers, two are "not strong enough"; two are "unsuitable for nursing"; two "for private reasons"; one to "go in for medicine"; one "to go home"; and one "giving up nursing." Of two staff nurses, one resigned "to take a better post," and the other "to take up private nursing." A dispenser left because the "dispensary was too hot on account of steam radiator."

---

How few comparatively have expressed in practical form their sense of thankfulness that victory has been won! A notable lead has been given by Mr. Samuel Megitt Johnson, of Endcliffe Court, who has presented a gift of £5,000 to the Sheffield Queen Victoria Nursing Association, "as a tangible expression of gratitude to Almighty God that the war is ended." Mr. Johnson has always taken a deep interest in district nursing and has chosen this channel for his gift because he is conscious that the Association is doing good work for the poorer people of Sheffield, and could do more if funds permitted. The money is to be invested in war stock and ultimately used to purchase a new home for the Association with grounds which would be of immense benefit to the nursing staff. The house to house collection for the Association, undertaken just before Christmas, has realised £770.

---

The much-desired new Nurses' Home at the Chelsea Hospital for Women has benefited to the extent of £220 by the concert and sale of work recently organised by the matron and nursing staff of the hospital. This is an admirable result which testifies to the able manner in which all worked together beforehand. We are very glad to learn that the sum of £7,000 is now in hand, and we trust the new Home is within measurable distance.

---

At the annual meeting of the Essex Branch Council of the British Red Cross Society the County Director, Mr. E. J. Wythes, gave an interesting summary of the work carried through during the war. Close on 40,000 patients passed through their hospitals which are now all closed. The total amount raised in the county for the period of the war was £26,463. Twelve motor ambulances have been allotted to Essex for the benefit of the civilian population, and there are still 2,714 members of detachments ready for any work which offers.

---

There will be a meeting on March 8 at Beechwood, Hampstead Lane, the residence of Mrs. E. Perronet Sells, O.B.E., for the purpose of furthering the work of the Ladies' Association connected with the Great Northern Central Hospital and enlisting new recruits. This is the Jubilee year of the hospital, and great efforts are being made to broaden the basis of support and attract all within the sphere of its operations to lend their aid. The Marquis of Northampton, Chairman of the hospital, will speak, and also the Bishop of Willesden, Lady Sargant, Chairman of the Ladies' Association, and Mr. G. B. Mower White, F.R.C.S.

